# Carajurin Induces Apoptosis in *Leishmania amazonensis* Promastigotes through Reactive Oxygen Species Production and Mitochondrial Dysfunction

**DOI:** 10.3390/ph15030331

**Published:** 2022-03-09

**Authors:** João Victor Silva-Silva, Carla J. Moragas-Tellis, Maria S. S. Chagas, Paulo Victor R. Souza, Davyson L. Moreira, Daiana J. Hardoim, Noemi N. Taniwaki, Vanessa F. A. Costa, Alvaro L. Bertho, Daniela Brondani, Eduardo Zapp, Aldo Sena de Oliveira, Kátia S. Calabrese, Maria D. Behrens, Fernando Almeida-Souza

**Affiliations:** 1 Laboratory of Immunomodulation and Protozoology, Oswaldo Cruz Institute, Oswaldo Cruz Foundation, Rio de Janeiro 21040-900, Brazil; jvssilva89@gmail.com (J.V.S.-S.); hardoim@ioc.fiocruz.br (D.J.H.); fernandoalsouza@gmail.com (F.A.-S.); 2 Laboratory of Natural Products for Public Health, Pharmaceutical Technology Institute, Farmanguinhos, Oswaldo Cruz Foundation, Rio de Janeiro 21040-900, Brazil; carla.tellis@far.fiocruz.br (C.J.M.-T.); msocchagas@gmail.com (M.S.S.C.); pvrs.pvrs@gmail.com (P.V.R.S.); dmoreira@far.fiocruz.br (D.L.M.); mariabehrens@hotmail.com (M.D.B.); 3 Postgraduate Program in Translational Research in Drugs and Medicines, Farmanguinhos, Oswaldo Cruz Foundation, Rio de Janeiro 21040-900, Brazil; 4 Electron Microscopy Nucleus, Adolfo Lutz Institute, Sao Paulo 01246-000, Brazil; ntaniwak@hotmail.com; 5 Laboratory of Immunoparasitology, Oswaldo Cruz Institute, Oswaldo Cruz Foundation, Rio de Janeiro 21040-900, Brazil; vanessaabreu@aluno.fiocruz.br (V.F.A.C.); alvaro.bertho@hotmail.com (A.L.B.); 6 Flow Cytometry Technological Platform, Oswaldo Cruz Institute, Oswaldo Cruz Foundation, Rio de Janeiro 21040-900, Brazil; 7 Research Group on Medicinal and Biological Chemistry (GPQMedBio), Department of Exact Sciences and Education, Federal University of Santa Catarina, Blumenau 89036-002, Brazil; daniela.brondani@ufsc.br (D.B.); eduardo.zapp@ufsc.br (E.Z.); aldo.sena@ufsc.br (A.S.d.O.); 8 Postgraduate Program in Animal Science, State University of Maranhão, Sao Luis 65055-310, Brazil

**Keywords:** carajurin, *Leishmania amazonensis*, mitochondria, cell death, apoptosis

## Abstract

Carajurin is the main constituent of *Arrabidaea chica* species with reported anti-*Leishmania* activity. However, its mechanism of action has not been described. This study investigated the mechanisms of action of carajurin against promastigote forms of *Leishmania amazonensis*. Carajurin was effective against promastigotes with IC_50_ of 7.96 ± 1.23 μg.mL^−1^ (26.4 µM), and the cytotoxic concentration for peritoneal macrophages was 258.2 ± 1.20 μg.mL^−1^ (856.9 µM) after 24 h of treatment. Ultrastructural evaluation highlighted pronounced swelling of the kinetoplast with loss of electron-density in *L. amazonensis* promastigotes induced by carajurin treatment. It was observed that carajurin leads to a decrease in the mitochondrial membrane potential (*p* = 0.0286), an increase in reactive oxygen species production (*p* = 0.0286), and cell death by late apoptosis (*p* = 0.0095) in parasites. Pretreatment with the antioxidant NAC prevented ROS production and significantly reduced carajurin-induced cell death. The electrochemical and density functional theory (DFT) data contributed to support the molecular mechanism of action of carajurin associated with the ROS generation, for which it is possible to observe a correlation between the LUMO energy and the electroactivity of carajurin in the presence of molecular oxygen. All these results suggest that carajurin targets the mitochondria in *L. amazonensis*. In addition, when assessed for its drug-likeness, carajurin follows Lipinski’’s rule of five, and the Ghose, Veber, Egan, and Muegge criteria.

## 1. Introduction

Leishmaniasis is one of the world’s most neglected diseases, with more than 1 billion people living at risk of infection in around 92 countries or territories where the disease is considered endemic [1]. Leishmaniasis treatment is not totally successful and vaccine candidates in humans still need to be evaluated by further clinical trials [2]. The available chemotherapeutic options have serious limitations, such as high costs, limited efficacies, and high toxicities. In addition, prolonged parenteral administration hinders patient adherence to treatment, impacting the appearance of drug-resistant strains [3].

Acknowledging the need to overcome the limitations of anti-leishmanial chemotherapy, plant-derived natural products have shown promising results for new antiprotozoal therapies, due to their vast chemical diversity. These products may be useful as an alternative and safe approach against leishmaniasis [4,5].

Mitochondria play a central role in generation cellular energy production and the survival of any cell depends on the proper function of these organelles [6]. The fact that kinetoplastids have a single mitochondrion indicates that this organelle is a potential candidate for the development of drugs [7]. The mitochondrial ultrastructural changes of *Leishmania* are associated with apoptosis-like death by the potential impairment of the mitochondrial membrane and/or by reactive oxygen species (ROS) production [8].

*Arrabidaea chica* (Humb. & Bonpl.) B. Verlot, a plant popularly known as crajiru that is native to the Amazon rainforest [9], has been used in folk medicine for wound healing, treatment of inflammation, and antioxidant activities, possibly related to the presence of anthocyanidins [10]. Analyzing different morphotypes of *A. chica*, we observed that its hydro-alcoholic extract is rich in anthocyanidins, mainly carajurin [11]. We recently demonstrated, through the bioguided fractionation of *A. chica* extract, that carajurin favored its leishmanicidal activity [12], validating our earlier identification of carajurin as a pharmacological marker for the anti-leishmanial potential of *A. chica*. However, the mechanism of action of carajurin on *L. amazonensis* has not been previously studied. Thus, this work aimed to demonstrate the mechanisms involved with cell death induced by carajurin in promastigote forms of *L. amazonensis*.

## 2. Results

### 2.1. Phytochemical Analysis

**Figure 1:**
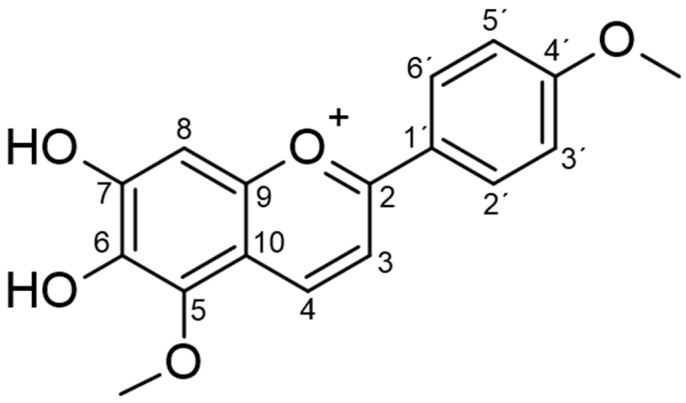
Chemical structure of carajurin.

The molecular formula C_17_H_15_O_5_ was established by the positive mode quasi-molecular ion peaks at m/z 299.0905 for [M + H]^+^ (calcd. 299.0924 for C_17_H_15_O_5_) in combination with its NMR data as previously described [12]. Carajurin purity was previously determined by HPLC-DAD-UV as 98%, as well as by data from ESI-MS experiments and by NMR ^1^H and ^13^C data [11]. The compound was initially described by Chapman et al. [13] and characterized by Zorn et al. [14] and Devia et al. [15]. Unequivocal characterization of carajurin (Table 1; Figure 1) was confirmed by other structural experiments, such as distortionless enhancement by polarization transfer (DEPT) and NMR-2D, homonuclear correlated spectroscopy (COSY), heteronuclear multiple bond coherence (HMBC), and heteronuclear single quantum correlation (HSQC) (Supplementary Materials Figures S1–S4). The ^1^H NMR, together with HMBC spectra, confirmed the presence of two methoxyl groups [δH 4.10 (s) and 3.90 (s)] that showed correlation with the aromatic carbons at δ162.50 and d 135.02, respectively, which were assigned as carbons 4′and 5. Protons at δ7.89 and 7.01 ppm could be attributed to the aromatic protons of B rings H2′/H6′ and H3′/H5′, due to the correlations observed with C2′/C6′(δ127.68) and C3′/C5′(δ114.77) at the HSQC spectra. The position of protons 3 and 4 was confirmed by the correlations observed, respectively, with the H4 and H3 in the COSY spectrum, and by the observed correlations of the HMBC spectra for H3 long-distance interactions with C10, C1′, and C2, and for H4 with C5, C2, and C9.

### 2.2. Anti-leishmanial Activity and Cytotoxicity

**Figure 2:**
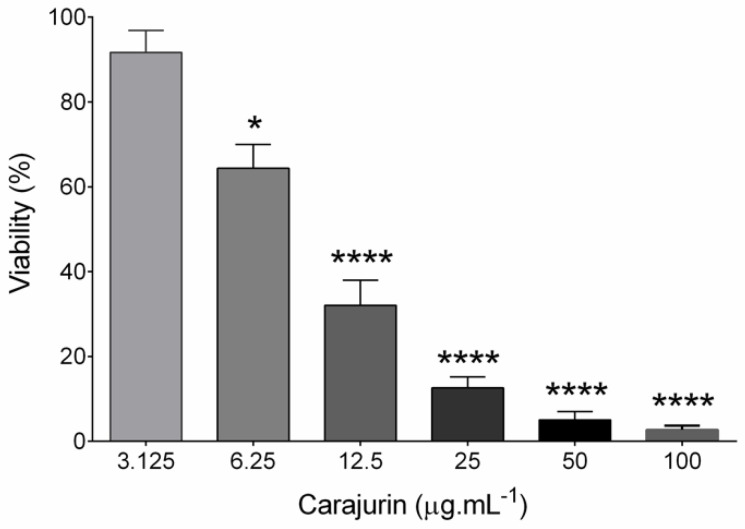
Effects of carajurin on growth of *Leishmania amazonensis* promastigote forms. Parasites in log-phase (2 × 10^6^ mL^−1^) were incubated in different concentrations of carajurin. Data represent the mean ± standard error of three independent experiments carried out in triplicate. (*) *p* < 0.05; (****) *p* < 0.0001, when compared to untreated parasites by Mann-Whitney test.

The anti-leishmanial activity of carajurin was evaluated in promastigote forms of *L. amazonensis*. Viable promastigotes were counted in a Neubauer chamber according to Rottini et al. [16], with the percentage of growth inhibition calculated from the count of viable parasites in relation to the untreated control to determine the values of 50% of inhibitory concentration (IC_50_). The results showed a significant concentration-dependent decrease (*p* < 0.0001) in parasite viability (Figure 2), with IC_50_ at 7.96 ± 1.23 μg.mL^−^^1^ (26.4 µM). In the evaluation of carajurin against peritoneal macrophage cells, the CC_50_ was approximately 33-fold higher when compared to the IC_50_ (Table 2), indicating that carajurin was more toxic to the parasites than to the cells. Amphotericin B was active against the promastigotes and peritoneal macrophages.

### 2.3. Ultrastructural Changes

**Figure 3:**
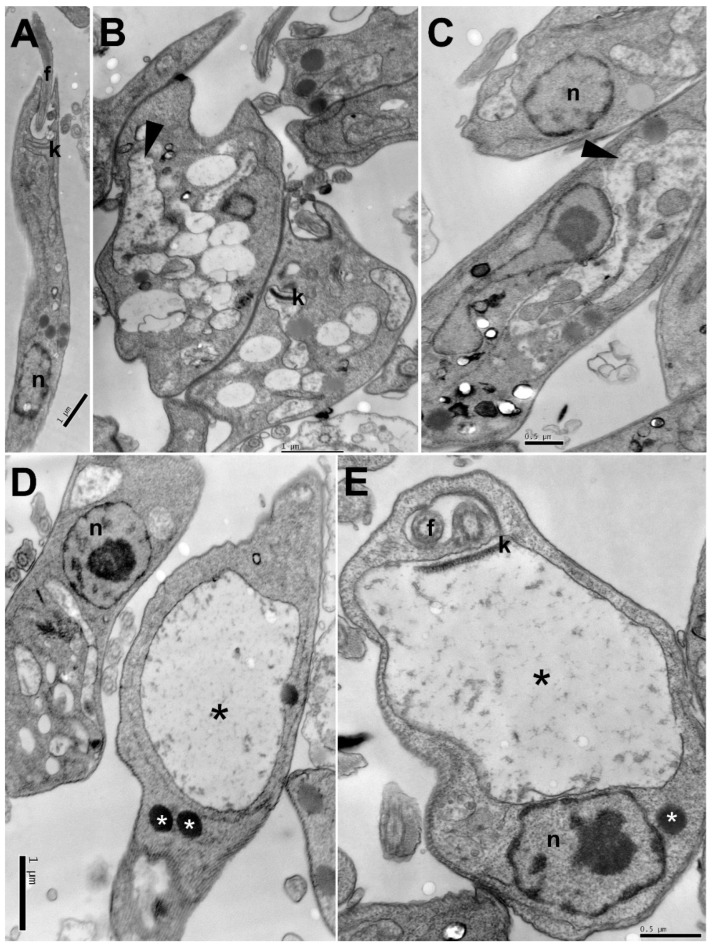
Ultrastructural changes in promastigote forms of *Leishmania amazonensis* treated with carajurin: (**A**) untreated parasites; (**B**–**E**) parasites treated for 24 h with carajurin (IC_50_). Electron-dense corpuscles (white asterisks), vesicles with electron-dense content (arrowhead), kinetoplast swelling (black asterisks). N = nucleus, f = flagellum, k = kinetoplast.

Transmission electron microscopy analyses were performed to evaluate ultrastructural alterations caused on *L. amazonensis* promastigote forms treated with the IC_50_ of carajurin. Figure 3A shows the well-preserved cell morphology of nontreated parasites, with characteristic elongated fusiform shape, and all its organelles with typical morphology. Figure 3B–E shows numerous and large vesicles in cytoplasm, some of them with electron-dense content, electron-dense corpuscles, and pronounced swelling of the kinetoplast with loss of electron-density.

### 2.4. Mitochondrial Membrane Potential (Δψm)

**Figure 4:**
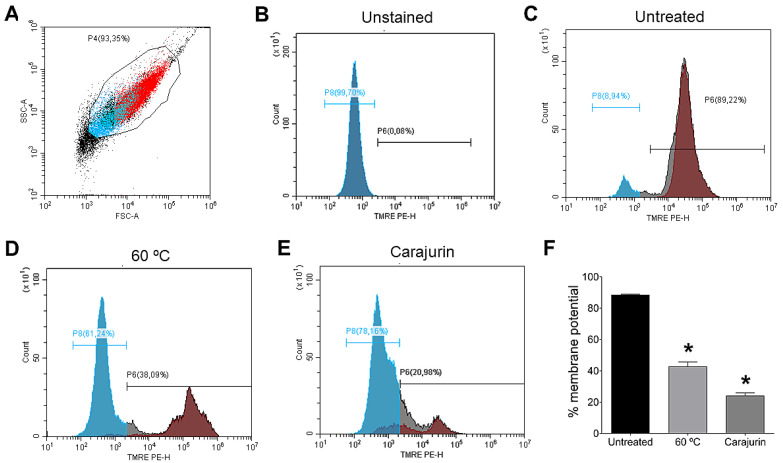
Flow cytometry of *Leishmania amazonensis* treated with IC_50_ of carajurin to assess the potential of the mitochondrial membrane (∆Ψm). (**A**) Promastigotes captured in the gated region and representative histogram. (**B**) Unstained parasites. (**C**) Untreated parasites. (**D**) Promastigote forms of *L. amazonensis* killed by heat. (**E**) Histogram representative of promastigotes treated with carajurin. (**F**) Statistically significant differences were observed between the percentages of cells marked with TMRE in the untreated group and the groups treated with carajurin, at the IC_50_ concentration (26.4 µM). (*) *p* < 0.05, when compared with the untreated group by Mann-Whitney test.

Mitochondrial membrane potential (Δψm) plays a key role in vital mitochondrial functions, as it is directly linked to ATP synthesis and, as such, its regulation is essential for cell viability. As mitochondria damage was observed in the ultrastructural analysis, flow cytometry analysis was performed to confirm carajurin-induced damage to the mitochondria. Flow cytometric analysis used tetramethylrhodamine ethyl ester (TMRE), a cell-permeant fluorescent dye that is readily sequestered by active mitochondria. Statistically significant changes in mitochondrial membrane potential were observed after treatment with carajurin (*p* = 0.0286). TMRE labeling after 24 h-treatment with carajurin was 20.98 ± 3.70%, while non-treated parasites’ labeling was 89.22 ± 0.67% (Figure 4).

### 2.5. Measurement of ROS

**Figure 5:**
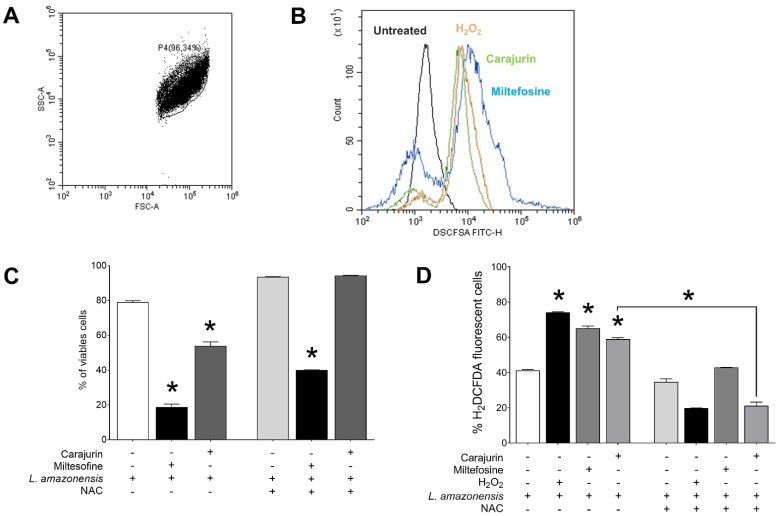
Evaluation of intracellular ROS levels in *Leishmania amazonensis* promastigotes and effect of N-Acetyl-L-cysteine on carajurin-induced cell death after incubation with carajurin for 24 h. (**A**) Promastigotes captured in the gated region and representative histogram. (**B**) The green line shows increased ROS production in parasites treated with carajurin, at the IC_50_ concentration (26.4 µM), when compared to control parasites (black line). The same was observed in the group treated with H_2_O_2_ (orange line) and miltefosine (blue line). (**C**,**D**) Promastigotes were cultivated in the presence of N-Acetyl-L-cysteine (NAC, 300 µM) and carajurin (IC_50_ concentration, 26.4 µM). Miltefosine (50µM) was used as a cell death control, and H_2_O_2_ (50 μM) as a natural inducer of ROS. Generation of cell death and ROS was measured using fluorescent dye propidium iodide (PI) and H_2_DCFDA, respectively. (*) *p* < 0.05, when compared to untreated parasites by Mann-Whitney test.

To investigate whether the leishmanicidal effect of carajurin is due to the production of ROS in *L. amazonensis* promastigote forms, ROS levels were measured using the cell-permeable dye H_2_DCFDA. Carajurin induced ROS production in parasites, with ROS levels increased to 58.9 ± 1.65% (*p* = 0.0286), in comparison to untreated parasites (Figure 5). Hydrogen peroxide (H_2_O_2_) and miltefosine were used as positive controls and resulted in increased ROS levels to 74.1 ± 0.86% (*p* = 0.0286) and 65.1 ± 3.11% (*p* = 0.0286), respectively. Furthermore, we evaluated whether the pre-incubation of *L. amazonensis* promastigotes with NAC could prevent the inhibitory effect of carajurin, and it was observed that NAC protected promastigotes from carajurin anti-leishmanial activity, enhancing the percentage of viable parasites (Figure 5C) by the reduction of the levels of ROS (Figure 5D).

### 2.6. Evaluation of Phosphatidylserine Exposure and Cell Membrane Integrity

**Figure 6:**
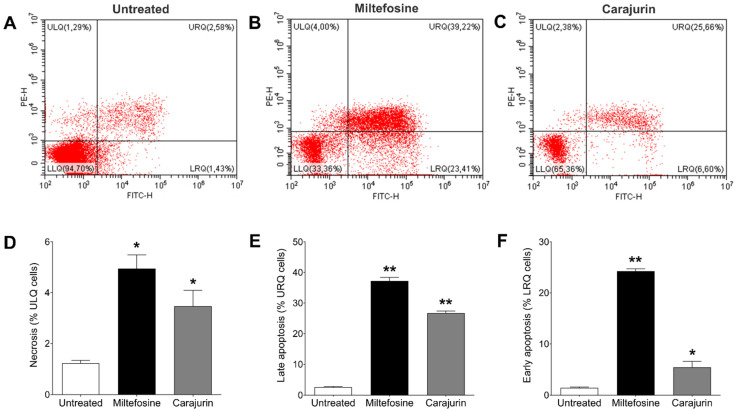
Changes in phosphatidylserine exposure and plasma membrane integrity in *Leishmania amazonensis* promatigotes treated with IC_50_ of carajurin for 24 h. Parasites were labeled with Annexin V-FITC and PI. (**A**) Untreated promastigotes used as control. (**B**) Parasites treated with miltefosine, an apoptosis-inducing drug, at a concentration of 50 μM. (**C**) Promastigotes treated with carajurin, at the IC_50_ concentration (26.4 µM). In (**D**–**F**), statistical differences between the percentage of necrotic, late apoptotic, and early apoptotic cells, respectively, are shown. In all, the results obtained in the groups treated with carajurin and miltefosine were statistically different when compared to untreated parasites. (*) *p* < 0.05; (**) *p* < 0.01, when compared to untreated parasites by Mann-Whitney test. ULQ: = upper left quadrant; URQ = upper right quadrant; LRQ = low right quadrant.

To determine the mechanism of cell death triggered by carajurin, promastigote forms were evaluated using Annexin V-FITC and PI staining to distinguish the necrotic or late apoptotic cells from the early apoptotic ones. After treatment with carajurin for 24 h, promastigotes captured in the closed region and the representative histogram (Figure 6A), as well as unstained parasites (Figure 6B), were observed, and the number of viable parasites decreased from 94.7 ± 3.32% to 65.36 ± 0.56% (lower left quadrant; *p* = 0.0238, Figure 6C). The percentage of parasites staining positive for PI but negative for Annexin V (upper left quadrant, Figure 6C) increased to 2.38 ± 1.53% (*p* = 0.0159, Figure 6D), and the intensity of Annexin-V and PI fluorescence (upper right quadrant) increased up to 25.66 ± 1.54% (*p* = 0.0095, Figure 6E), compared to untreated parasites. Carajurin also induced early-stage apoptosis with the percentage increased to 6.60 ± 2.52% (*p* = 0.0119, Figure 6F) (lower right quadrant). These results suggest that carajurin induces late apoptosis in *L. amazonensis* promastigotes.

### 2.7. Electrochemical Tests/Cyclic Voltammetry

**Figure 7:**
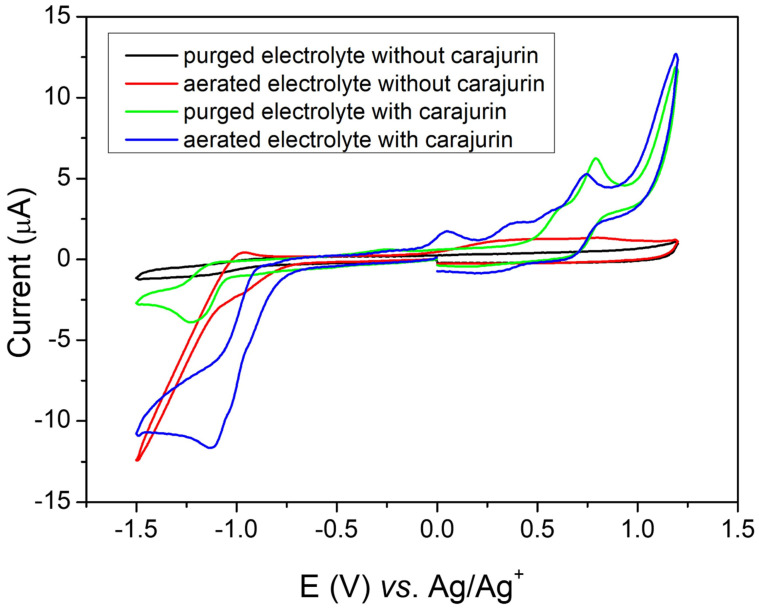
Cyclic voltammograms obtained at glassy carbon electrode in dichloromethane containing 0.05 mol L^−1^ of TBAPF_6_ (electrolyte) at scan rate of 50 mV s^−1^, where the black line shows purged electrolyte (without oxygen) in the absence of carajurin, the red line shows aerated electrolyte (with oxygen) in the absence of carajurin, the green line shows electrolyte with carajurin in the absence of oxygen, and the blue line shows electrolyte with carajurin in the presence of oxygen.

The potential was applied to the working electrode at a constant rate (50 mV s^−1^), and for evaluation of oxidizing species the potential was swept in the window from 0 to 1.2 V vs. Ag/Ag^+^. To evaluate the reduction processes, the potential was swept in the window from 0 to −1.5 V vs. Ag/Ag^+^). Carajurin presented the first oxidation process at 0.47 V vs. Ag/Ag^+^ (0.42 V vs. NHE) (Figure 7, green line), which can be attributed to the oxidation of the hydroxy substituent; the second oxidation peak is probably due to the formation of an intermediate radical produced during the oxidation process. Both oxidation processes showed some reversibility; however, the cathodic peaks showed slower processes, resulting in lower peak currents [17]. As shown in Figure 7 (green line), when scanning at more negative potentials, the carajurin in oxygen absence showed a reversible process in potential,1.07 V vs. Ag/Ag^+^ (–1.12 V vs. NHE), which indicated the compound’s ability to capture electrons forming a radical anion, a factor that influences in the compound’s properties [18,19]. Figure 7 (red line) shows the voltammetric response in an air-saturated electrolyte solution, associated with superoxide radical generation, in the absence of carajurin. The possible interaction of the radical anion with oxygen was studied from the carajurin response in the presence of dissolved oxygen (Figure 7, green line), showing a shift from the reduction process to more positive potential values. The energies of HOMO and LUMO were calculated from the first oxidation and the reduction process, respectively. The experimental energy values of the HOMO and LUMO levels for carajurin were –4.86 eV and –3.32 eV, respectively; therefore, the gap estimated from the electrochemical data was 1.54 eV.

### 2.8. Quantum Studies

**Figure 8:**
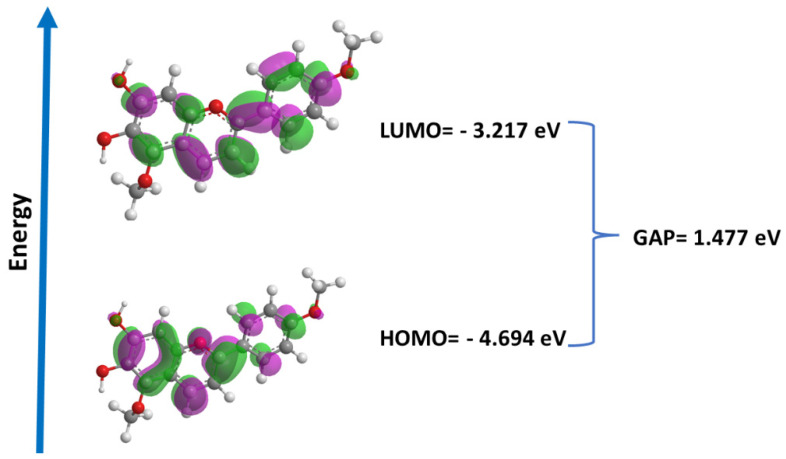
Energies and surfaces of the frontier orbitals (LUMO and HOMO) and GAP (LUMO-HOMO) for carajurin.

Molecular orbitals play a crucial role in understanding chemical reactivity at the atomic level and are important descriptors for the rationalization of various chemical reactions, in addition to comprising a wide range of biological activities. In this study, we performed the calculation of the energies of the frontier orbitals (LUMO and HOMO) for carajurin (Figure 8), with results that are compatible with the observations from the cyclic voltammetry experiments.

### 2.9. In Silico Prediction Physico-Chemical

To analyze the profile of cajurin as a prototype for drugs in preclinical stages of development, the Swiss ADME^®^ platform (http://www.swissadme.ch/index.php, accessed on 16 February 2022) was used, from which were extracted information about: physicochemical properties, Drug-likeness, and Medicinal chemistry (Table 3).

## 3. Discussion

Previous studies demonstrated a direct effect of *A. chica* against *L. amazonensis* and *L. infatum* promastigote forms [20,21]. Our research group reported that *A. chica* was able to inhibit the proliferation of promastigote forms of *L. amazonensis* [11,12,22]. Moreover, we verified a superiority of carajurin among the other anthocyanidins in inhibiting *Leishmania* promastigotes, demonstrating the direct action of this natural compound on this parasite [12]. Data described in the previously published article [12], emphasize the inhibition of intracellular amastigote forms, indicating activation of leishmanicidal macrophage functions, especially the induction of NO. These results showed that carajurin can act indirectly by activation of mechanisms in macrophage, such as an increased release of NO. However, in the present study we discuss a possible mechanism of inhibition against the free forms of the parasite (extracellular evolutionary form, promastigote), so the data presented here refer to the direct effect on the parasite.

Carajurin stood out as the most active anthocyanidin, with IC_50_ less than 4 μg.mL^−1^ after 72 h of treatment [12]. However, in the present study, using a 24-h treatment, an increase in selectivity by 7 times (SI = 32.4) was observed, compared to the 72-h treatment. Compounds with SI ≥ 10 are considered effective anti-leishmanial compounds, compared with in vitro cytotoxicity [23]; thus, carajurin deserves to be considered as a good candidate for further experimental chemotherapy studies against *Leishmania*.

Observations of ultrastructural and morphological alterations are used to elucidate the mechanisms of action of new compounds and to investigate the cell death mechanism involved [24]. Consequently, to investigate and identify which organelles are the targets of carajurin and the damages within the parasite, ultrastructural analyses of *L. amazonensis* promastigotes were performed using transmission electron microscopy. In promastigotes treated for 24 h with the IC_50_ of carajurin, we observed vesicles with electron-dense content, electron-dense corpuscles, and pronounced swelling of the mitochondria.

There is little information available on the leishmanicidal activity of anthocyanidins for comparison with the results presented herein. Previous studies by our group suggested that the leishmanicidal activity of carajurin would be associated with the ability to induce the activation of the microbicidal response in macrophages and promote the production of NO [12]. Furthermore, data from the literature report that flavonoids can target the kinetoplast of parasites, as they induce significant cleavage of the topoisomerase II-mediated kDNA minicircle in *Leishmania* [25].

Important alterations on the ultrastructure of *L. infantum* promastigotes were also observed in parasites treated with the fraction obtained with an increasing gradient of polarity (hexane:ethyl acetate) from *A. chica* hexane extract [20]. In that study, parasites exposed to the active fraction (18.6 μg/mL, 24 h) showed abnormal cell body shapes. Mitochondrial dilatation with loss of matrix contents and Golgi complex alterations, followed by a cytoplasm vacuolization process and an intense exocytic process of cytoplasmic content into the flagellar pocket, were also observed [20]. The experiments with dimeric flavonoid (braquidina 2) from *Arrabidaea* *brachypoda* also demonstrated alterations in the Golgi complex and the accumulation of vesicles inside the flagellar pocket in *L. amazonensis* amastigotes [26]. In addition, other drugs directly interfere with mitochondrial physiology in parasites such as *Leishmania* [27,28]. The mitochondria of protozoan are considered an ideal drug target, while minimizing toxicity [6,29]. Anti-trypanosomal compounds, such as pheophorbide A [30] obtained from leaves of *A. chica*, also affected the parasite’s mitochondrion. The ultrastructural alterations induced by pheophorbide A in trypomastigotes of *T. cruzi* were similar to those observed in the present study for carajurin in *L. amazonensis*. These results suggest mitochondrial collapse as part of the mechanism of action of carajurin and demonstrate its leishmanicidal effect.

To confirm drastic damage to the mitochondrion of the parasite evidenced by transmission electron microscopy, the ΔΨm was evaluated by flow cytometry using TMRE. We observed that carajurin induced the depolarization of the mitochondrial membrane of the promastigote parasite, showing that this compound is capable of crossing the plasma membrane and causing a collapse of the mitochondrial membrane of the parasite. Several plant compounds that cause mitochondrial damage and parasite death have their mechanisms of action attributed mainly to the potential dysfunction of the mitochondrial membrane [20,31].

Knowing that the production of ROS in promastigotes is one of the possible events triggered by the loss of mitochondrial integrity [32,33], we investigated whether carajurin could act through this process. Treatment of *Leishmania* promastigotes with carajurin resulted in a significant increase of ROS levels and demonstrated that N-acetylcysteine (NAC) protected *L. amazonensis* from inhibition by carajurin, in addition to reducing the ROS levels in carajurin-treated cells. NAC is a thiol compound that increases the levels of glutathione [34]; it is an important molecule for protecting kinetoplastids from ROS or toxic compounds, acting as an antioxidant [35]. This result indicated that the inhibition of growth promoted by carajurin in *L. amazonensis* is mediated by ROS production. This might explain the depolarization of the mitochondrial membrane for this parasite stage. Studies conducted by Fonseca-Silva, et al. [6] reported that the mitochondrial dysfunction observed in *L. amazonensis* promastigote treated with the flavonoid quercetin is promoted by ROS production, in the same way as *L. amazonensis* promastigote treated with the flavonoid apigenin [36] is promoted, for the same parasites. Furthermore, the results of these compounds suggest the involvement of ROS in leading to an alteration of the mitochondrial membrane potential as part of the mechanism of action.

Mitochondrial ROS production followed by the depolarization of the mitochondrial membrane can trigger parasite death through an apoptosis-like mechanism [29,32,37,38]. Promastigotes of *L. amazonensis* treated with carajurin IC_50_ were double stained with Annexin V and PI to evaluate cell death induction, and it was observed that carajurin induced late apoptosis in parasites.

Similar to our findings, previous studies reported cell death induction in *L. amazonensis* promastigotes induced by compounds isolated from natural products [39,40]. In addition, luteolin and quercetin inhibited DNA synthesis in *L. donovani* promastigotes and promoted topoisomerase-II mediated linearization of kDNA minicircles, leading to apoptosis [25]. In other studies, the flavonoids fisetin, quercetin, and luteolin inhibited the arginase enzyme from *L. amazonensis* [41,42]. L-arginine deprivation promotes an externalization of phospholipids that bind to Annexin V, signaling apoptosis-like cell death in *L. donovani* promastigotes [43].

Cyclic voltammetry (CV) is a simple method for screening active redox compounds and estimating electrochemical activity in different samples, such as medicinal plants [17]. To analyze the mode of action of carajurin, the electrochemical behavior of the compound was analyzed by CV in an aprotic organic environment (dichloromethane), in order to mimic the nonpolar cellular environment [18,19]. Under aerobic conditions, the compound reduction mechanism predominates, resulting in the radical anion intermediate, which, when undergoing a retro-oxidation process in the presence of oxygen, releases ROS, similar to the process described for other compounds in actions against parasites [18,19,44,45]. When analyzing the electroactivity of carajurin, it can be observed that in the absence of molecular oxygen (Figure 7, green line) the compound shows a reduction process, which can lead to the generation of the radical intermediate. As seen in Figure 7 (blue line), in the presence of molecular oxygen, the voltammogram shows a change in the profile, indicating a possible interaction of carajurin electroreduction products with dissolved oxygen in the electrochemical cell [18,19]. These effects include a shift from the position of the peak of carajurin reduction, for more positive potentials.

As the electronic level is an inherent characteristic of a substance, the redox potential is also a unique value of the substance; thus, the electrochemical data obtained by voltammetry were used to determine the energy of the boundary orbital, which data were similar to the values obtained by DFT. HOMO energy is a better indicator of antioxidant activity than LUMO energy; in general, it is possible to relate HOMO energy values and scavenging activities. On the other hand, the energy of LUMO is a better indicator in relation to antiparasitic activity, as it is associated with the molecule’s reduction process. Furthermore, the HOMO and LUMO values obtained from voltammetry and quantum studies suggest that carajurin can acquire an electron more easily than donating an electron, favoring its action in the generation of ROS over an antioxidant action, in agreement with the results obtained in computational studies [46].

From the data in Table 3, it is possible to observe that carajurin has a set of physicochemical properties (molecular weight, rotational bonds, H-bond acceptors, H-bond donors, surface area TPSA-Å2, and lipophilicity (log Po/w) according withwhat is expected for a drug, since it does not contradict any of the rules established by Lipinski, Ghose, Veber, Egan, and Mueggue. Furthermore, carajurin did not present pan-assay interference compounds (PAINS). These data encourage further research with carajurin with an in-depth analysis of pharmacokinetic parameters (ADME) and toxicity, using a combination of in silico and in vitro strategies. Finally, these results advance our knowledge on the mechanisms involved in the leishmanicidal effect of carajurin, building solid foundations for drug discovery and opening new opportunities for research in this significant area of human health.

Taken together, our results are consistent with findings with respect to *L. amazonensis* promastigotes, that the presence of ROS causes mitochondrial depolarization and that this can trigger parasite death through an apoptosis-like mechanism. In addition, further in silico and in vitro enzymatic evaluation tests of carajurin are being developed to achieve a better understanding of the mechanism by which carajurin acts in promoting a leishmanicidal effect.

## 4. Materials and Methods

### 4.1. Reagents

The reagents 2,7-dichlorodihydro-fluorescein (H_2_DCFDA), Brewer thioglycolate medium, RPMI 1640 medium, 3-(4,5-dimethylthiazol-2-yl)-2,5-diphenyltetrazolium bromide (MTT), dimethyl sulfoxide (DMSO), EPON 812 resin, glutaraldehyde, amphotericin B, osmium tetroxide, Schneider’s insect medium, and streptomycin were purchased from Sigma-Aldrich (St. Louis, MO, USA). Fetal bovine serum (FBS), and penicillin were acquired from Gibco (Gaithersburg, MD, USA). Tetramethylrhodamine ethyl ester (TMRE) was obtained from Molecular Probes (Carlsbad, CA, USA).

### 4.2. Plant Material

*Arrabidaea chica*, morphotype IV, were collected in February 2016 from the Fiocruz Atlantic Forest Campus, Rio de Janeiro city, State of Rio de Janeiro, Brazil (S 22.9406° W 43.4046°). The leaves were identified by Dr. Marcelo Galvão, and voucher specimens were deposited at the Botanical Collection of Medicinal Plants (CBPM) of Farmanguinhos/Fiocruz (CPBM 668).

### 4.3. Isolation and Structural Characterization of Carajurin

Carajurin was isolated as a red amorphous powder from *A. chica* hidroalcoholic extract after successive fractionation steps, such as liquid-liquid partition and column chromatography using Sephadex as the stationary phase and dichlorometane:metanol (1:1) as the eluent. The present work shows other structural characterization results of carajurin, in addition to those recently shown in a previous paper [12]. Distortionless enhancement by polarization transfer (DEPT) NMR-1D and NMR-2D as homonuclear correlated spectroscopy (COSY), heteronuclear multiple bond coherence (HMBC), and heteronuclear single quantum correlation (HSQC) were useful to confirm the unequivocal identification of anthocyanidin carajurin. NMR 1D and 2D analyses were recorded with a Bruker 400 (Wissembourg, France), 400.15 MHz (^1^H) and 100.62 MHz (^13^C). The chemical shifts were determined relative to CDCl_3_ at 0 ppm.

### 4.4. Ethical Statements and Animals

Female BALB/c mice aged 4 to 6 weeks were purchased from the Institute of Science and Technology in Biomodels of the Oswaldo Cruz Foundation. The Ethics Committee on Animal Care and Utilization reviewed and approved the animal protocol (CEUA-IOC L53/2016). All procedures described by the Control of Animal Experimentation (CONCEA) were strictly followed.

### 4.5. Peritoneal Macrophage Isolation and Parasite Cultures

Peritoneal macrophages were isolated from BALB/c mice administered with 3 mL thioglycolate 3% intraperitoneal for 72 h. Then, cells were cultured overnight and maintained in RPMI 1640 medium, at 37 °C and 5% CO_2_. *Leishmania amazonensis* strain MHOM/BR/76/MA-76 was maintained in promastigote form by culturing at 26 °C Schneider’s Insect Medium. All media were supplemented with 10% fetal bovine serum, 100 U mL^−1^ penicillin, and 100 μg mL^−1^ of streptomycin.

### 4.6. In Vitro Cytotoxicity Assay of Carajurin on Peritoneal Macrophages (CC_50_)

Cell viability was determined by 3-(4,5-dimethylthiazol-2-yl)-2,5-diphenyl tetrazolium bromide (MTT) assay. Peritoneal macrophages were plated in 96-well plates at 5 × 10^5^ cells.mL^−1^. After cell adherence, the medium was removed and replaced by carajurin (3.9–500 μg.mL^−^^1^) or amphotericin B (0.19–25 μg.mL^−1^), in a final volume of 100 μL per well, at 37 °C and 5% of CO_2_. The plates were incubated for 24 h at 37 °C in a humidified incubator with 5% CO_2_. Wells without cells were used as blank and wells with cells and 1% DMSO were used as controls. MTT (5 mg/mL) was added to each well in a volume equal to 10% of the total. After 2 h, the supernatant was completely removed and 100 μL of DMSO was added to each well to dissolve the formazan crystals. The absorbance was read on a spectrophotometer at a wavelength of 570 nm. Data were normalized according to the following formula: % survival = (Abs. sample-Abs. blank)/(Abs. control-Abs. blank) × 100 [47].

### 4.7. In Vitro Inhibition Assay of Carajurin on Promastigotes (IC_50_) and Selectivity Index

The susceptibility of promastigotes was carried out according to the method described by Silva-Silva, et al. [12]. Promastigote forms of *L. amazonensis* harvested at the log phase were seeded into 96-well flat-bottomed plates at 2 × 10^6^ parasites per well. Then, serial dilutions of carajurin (100–3.125 μg mL^−^^1^) were obtained. After diluting, the plates were incubated at 26 °C for 24 h. The plates were examined under an inverted microscope to assure the growth of the controls under sterile conditions, and viable promastigotes were counted in a Neubauer chamber [16]. Amphotericin B (2.5–0.07 μg mL^−1^) was used as the reference drug, while wells without parasites were used as blanks, and wells with parasites and DMSO 1% only were used as an untreated control. The experiments were conducted in triplicate. The percentage of growth inhibition was calculated from the count of viable parasites relative to the untreated control, and 50% inhibitory concentration (IC_50_) values were determined. The selectivity index (SI) was obtained from the ratio between the half-maximal cytotoxic concentration (CC_50_) for BALB/c peritoneal macrophages and the IC_50_ for promastigote.

### 4.8. Transmission Electron Microscopy

Promastigote forms of *L. amazonensis* were treated with an IC_50_ carajurin concentration for 24 h, according to the calculated index for carajurin. Nontreated parasites were used as a control. After 24 h-incubation at 26 °C, promastigotes were collected by centrifugation at 1500× *g* for 5 min. The parasites were fixed with 2.5% glutaraldehyde in a 0.1 M sodium-cacodylate buffer, pH 7.2, overnight. Then, the parasites were washed three times with the 0.1 M sodium-cacodylate buffer and postfixed in a solution containing 1% osmium tetroxide, 0.8% ferrocyanide, and 5 mM calcium chloride, washed in the 0.1 M sodium-cacodylate buffer, dehydrated in graded acetone, and embedded in EMbed 812 resin. Ultrathin sections were obtained from 100 nm cuts in Sorvall MT 2-B (Porter Blum) ultramicrotome (Sorvall, Newtown, CT, USA) stained with a 5% uranyl acetate aqueous solution and lead citrate (1.33% lead nitrate and 1.76% sodium citrate), and examined with a transmission electron microscope, JEM-1011 (JEOL, Tokyo, Japan), operating at 80 kV [8].

### 4.9. Determination of Mitochondrial Membrane Potential (MMP)(ΔΨm)

To measure the mitochondrial membrane potential, promastigote forms of *L. amazonensis* (2 × 10^6^ parasites mL^−1^) were treated with carajurin for 24 h with calculated IC_50_, in Schneider’s Insect Medium supplemented with 10% heat-inactivated fetal bovine serum, 100 U mL^−1^ penicillin, and 100 μg mL^−1^ streptomycin. Heat-killed parasites (60 °C bath for 30 min) were used as positive control and nontreated parasites were used as a negative control. Subsequently, the parasites were incubated for 30 min at 26 °C with 50 nM tetramethylrhodamine, followed by ethyl ester (TMRE) for 15 min at room temperature, and submitted to flow cytometric analysis through a CytoFLEX flow cytometer (Beckman Coulter Life Sciences, Inc., Brea, CA, USA). TMRE fluorescence was excited through a 488 nm-blue laser and their fluorescence was collected at 585/42 bandpass filter. CytExpert software version 2.1 (Beckman Coulter Life Sciences, Inc., Brea, CA, USA) was used for flow cytometric analyses.

### 4.10. Measurement of Reactive Oxygen Species (ROS)

The ROS production was evaluated using cell permeable oxidative fluorescent dye 2′,7′dichlorodihydrofluorescein diacetate (H_2_DCFDA). *Leishmania amazonensis* promastigotes (2 × 10^6^ parasites mL^−1^) were treated with a IC_50_ carajurin concentration for 24 h at 26 °C. ROS production was also monitored in *Leishmania* promastigotes pretreated with N-acetylcysteine (NAC, 300 μM) for 60 min, followed by treatment with carajurin (IC_50_ concentration). Hydrogen peroxide (50 μM) and nontreated parasites were used as positive and negative controls, respectively. Then, the parasites were centrifuged, washed with PBS, and incubated with 150 μL of H_2_DCFDA (5 μM) for 30 min in the dark, at room temperature. The H_2_DCFDA-fluorescence intensity was measured by flow cytometry [8].

### 4.11. Detection of L. amazonensis Apoptosis by Flow Cytometry

Following the 24 h treatment with carajurin (IC_50_) at 26 °C, *L. amazonensis* promastigotes apoptosis and necrosis were analyzed, phosphatidylserine (PS) externalization; and plasma membrane integrity, respectively, using annexin V-FITC and propidium iodide (PI)/Dead Cell Apoptosis Kit (Invitrogen™), according to the manufacturer’s instructions, and followed by flow cytometry analysis. After the incubation time, the parasites were centrifuged at 1500 rpm for 5 min at room temperature, washed in PBS, and resuspended in a 100 μL 1X annexin-binding buffer, 5 μL Annexin V and 1 μL PI (100 μg mL^−1^). Cell death was also monitored in *Leishmania* promatigotes after carajurin (IC_50_) treatment using only PI (1 μL, 100μg mL^−1^). As control procedures, we used miltefosine (50 μM) (antileishmanial reference drug) and untreated parasites. After 15 min incubation protected from light at room temperature, a 400 μL 1X annexin-binding buffer was added to each sample. For analytical purposes, promastigotes were classified according to their staining as apoptotic parasites (annexin V+; PIneg), late apoptotic/necrotic parasites (annexin V+; PI+), and viable parasites (annexin Vneg; PIneg).

### 4.12. Electrochemical Tests/Cyclic Voltammetry

The cyclic voltammetry (CV) measurements were taken using a potentiostat/galvanostat Autolab PGSTAT 204 (Metrohm). CV was performed using 1.6 × 10^−4^ mol L^−1^ carajurin and 0.05 mol L^−1^ tetra-n-butyl ammonium hexafluorophosphate (TBAPF_6_) in dichloromethane as the supporting electrolyte. The experiments were performed using a standard three-electrode cell with a glassy carbon electrode, a Pt-wire counter electrode, and Ag/Ag^+^ (AgNO_3_ 0.01 mol L^−1^ in acetonitrile) as the reference electrode. For experiments in the absence of oxygen, before each measurement the cell was deoxygenated by purging with argon. The energies of the highest occupied molecular orbital (E_HOMO_) and the lowest unoccupied molecular orbital (E_LUMO_) can be related to the potential of the first oxidation and the reduction process of the molecule, respectively. To obtain the values of E_HOMO_ and E_LUMO_, initially the oxidation-reduction potentials were recalculated for the NHE scale, using the redox pair ferrocene/ferrocene (0.45 V vs. Ag/Ag^+^) as an internal standard. Using the corrected potentials for the NHE scale and considering the potential of the NHE on the absolute scale equal to 4.44 eV, we calculated the values of E_HOMO_ and E_LUMO_ using the following empirical formulas [48]: E_LUMO_ = −(E^red^_onset_ + 4.44) eV and E_HOMO_ = −(E^oxi^_onset_ + 4.44) eV, where E^red^_onset_ and E^oxi^_onset_ are the onset potentials (vs. NHE) of reduction and oxidation, respectively.

### 4.13. Quantum Studies

DTF calculations to estimate all energy values of the highest occupied molecular orbitals (HOMO) and the lowest unoccupied molecular orbitals (LUMO performed on Gaussian v.09 program package with B3LYP level and 6–311++G(d,p) basis sets [16], were applied in these molecular systems’ gas phase, considering the singlet and neutral structures. The calculations were run subject to the grid method and the Slater exchange potential correlations. Next, the Hückel [49] method generated an initial estimate of molecular orbitals and electronic density. Subsequently, the convergence of the self-consistent field (SCF) [50] was determined by the restricted Hartree-Fock (RHF) algorithm, which was limited to 30 iteration cycles [51].

### 4.14. In Silico Prediction Physico-Chemical

The structure of carajurin was drawn using ChemDraw software (version Ultra 12.0, PerkinElmer Informatics, Waltham, MA, USA) and was converted into a single database file, SMILES. In silico prediction of physico-chemical properties was made using SwissADME, a tool to increase reliability [52].

### 4.15. Statistical Analyses

The statistical analyses were conducted using the statistical software GraphPad Prism^®^ version 7 (GraphPad Software Inc., San Diego, CA, USA). The numerical results were expressed as mean ± standard deviation and differences were considered significant when *p* < 0.05.

## 5. Conclusions

The results obtained in this study show that the lethal effect of carajurin on the promastigote forms of *L. amazonensis* was the result of ultrastructural changes, mitochondrial membrane potential decrease, and increased ROS production, which together induced cell death by late apoptosis. In this context, our work helps to achieve a better understanding of the mechanism of action of this anthocyanidin against *L. amazonensis*.

## Figures and Tables

**Table 1:** NMR 1D and 2D data for carajurin.

Position	^1^H ^a,b^ 400 MHz (δ) in ppm (CDCl_3_)	Cosy (H/h Correlation)	^13^C ^b^ 100 MHz (δ) in ppm (CDCl_3_)	DEPT	HSQC (H/C Correlation)	Hmbc (H/C Correlation)
2	-		158.90	Q	-	-
3	6.98 (*d*, J = 19.5 Hz)	H4	102.62	CH	C3	C10; C1′; C2
4	7.99 (*d*, J = 19.5 Hz)	H3	133.76	CH	C4	C5; C2; C9
5	-	-	135.02	Q	-	-
6	-	-	139.93	Q	-	-
7	-	-	176.82	Q	-	-
8	6.53 (*s*)	-	98.61	CH	C8	C10; C6; C9
9	-	-	156.86	Q	-	-
10	-	-	118.16	Q	-	-
1′	-	-	123.43	Q	-	-
2′/6′	7.89 (*d*) J = 22.3 Hz	H3′e H5′	127.68	CH	C2′/C6′	C2′; C6′; C2, C4′
3′/5′	7.01 (*d*) J = 22.2 Hz	H2′e H6′	114.77	CH	C3′/C5′	C3′; C5′; C1′, C4′
4′	-	-	162.50	Q	-	-
OCH_3_-5	4.10 (*s*)	-	60.42	CH3	OCH_3_-5	C5
OCH_3_-4`	3.90 (*s*)	-	55.58	CH3	OCH_3_-4′	C4′

^a^ Multiplicities and coupling constants in Hz are shown in parentheses. ^b^ Data previously presented in Silva-Silva et al. [12].

**Table 2:** Antileishmanial activity, cytotoxicity, and selectivity index of carajurin for 24 h of treatment.

Compounds	Peritoneal Macrophages	*L. amazonensis* Promastigotes	
	CC_50_ (μg mL^−1^)	IC_50_ (μg mL^−1^)	SI
Carajurin	258.2 ± 1.20 (856.9 µM)	7.96 ± 1.23 (26.42 µM)	32.4
Amphotericin B	8.740 ± 1.08 (9.458 µM)	0.0299 ± 1.18 (0.03236 µM)	292.3

Data represent mean ± SD. CC_50_: cytotoxic concentration for 50% of cells; IC_50_: inhibitory concentration for 50% of parasites. SI: selectivity index.

**Table 3:** Predicted physicochemical, drug-likeness, and medicinal chemistry properties for carajurin.

Property/Model Name	Carajurin
**Physico-chemical**	
Molecular weight	301.31
# Rotatable bonds	3
# H-bond acceptors	5
# H-bond donors	2
Surface area	126.520
TPSA (Å2)	75.99
Lipophilicity (log Po/w)	0.68
**Drug-likeness**	
Lipinski	Yes; 0 violation
Ghose	Yes
Veber	Yes
Egan	Yes
Muegge	Yes
**Medicinal chemistry**	
PAINS	0 alert
Brenk	1 alert: charged oxygen sulfur
Lead-likeness	Yes
Syntheticaccessibility	4.24

#: number, TPSA: topological polar surface area; PAINS: pan-assay interference compounds; MW: molecular weight.

## Data Availability

Data is contained within the article or supplementary material.

## Supplementary Materials

The following supporting information can be downloaded at: https://www.mdpi.com/article/10.3390/ph15030331/s1. Figure S1: ^1^H-^13^C Heteronuclear Multiple Bond Correlation (HMBC) Spectroscopy for Carajurin. Figure S2: Expansion of the ^1^H-^13^C Heteronuclear Multiple Bond Correlation (HMBC) Spectroscopy for Carajurin. Figure S3: ^1^H-^13^C Heteronuclear Single Quantum Correlation (HSQC) Spectroscopy for Carajurin. Figure S4: ^1^H-^1^H Homonuclear Correlation (COSY) Spectroscopy for Carajurin.
