# Supplementary material for: Carajurin Induces Apoptosis in Leishmania amazonensis Promastigotes through Reactive Oxygen Species Production and Mitochondrial Dysfunction
Source: Pharmaceuticals (Basel). 2022 Mar 9;15(3):331. doi: 10.3390/ph15030331 (PMC8948652; doi:10.3390/ph15030331)

Figure S1 –^{1}H-^{13}C Heteronuclear Multiple Bond Correlation (HMBC) Spectroscopy for carajurin

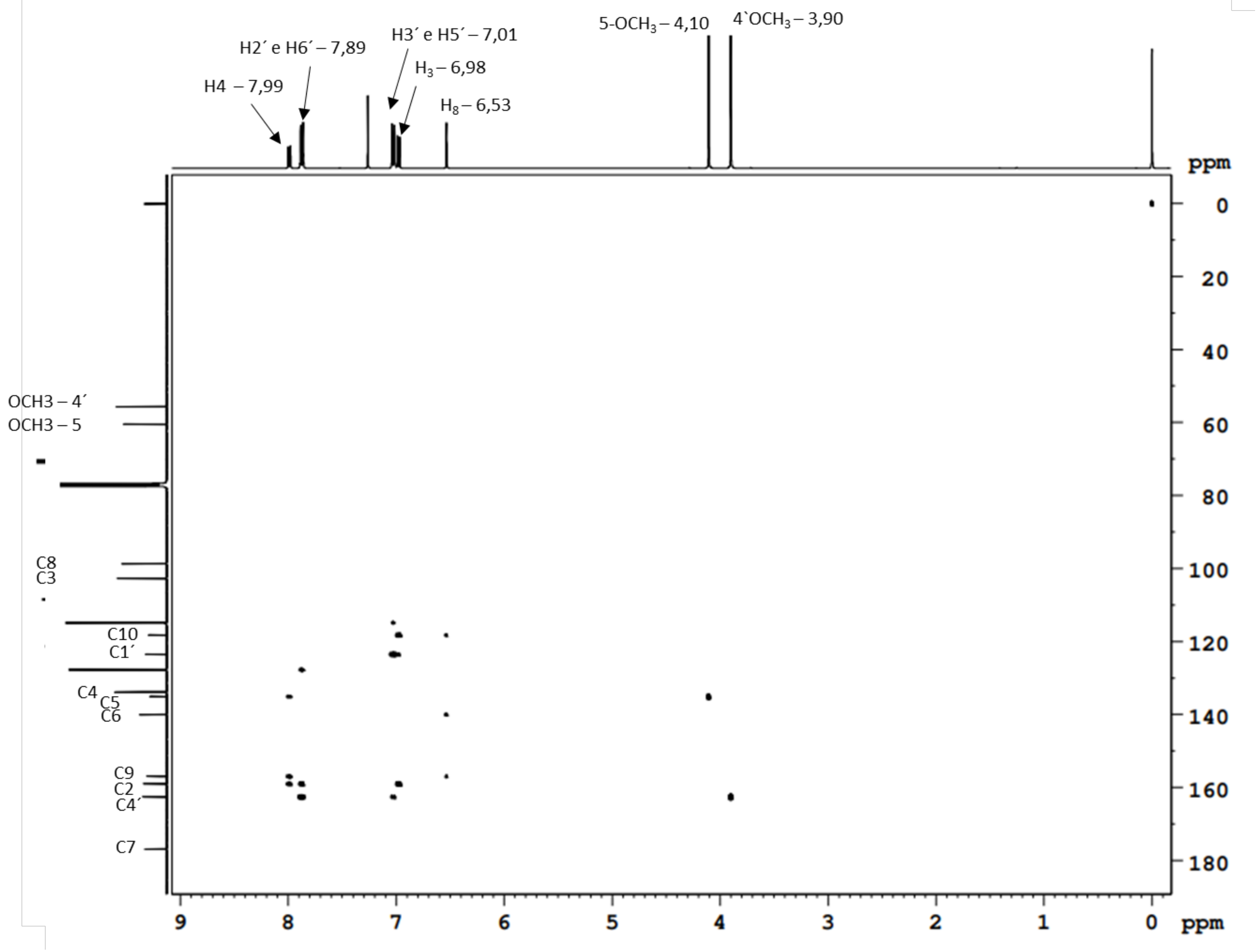

Figure S2 – Expansion of the ^{1}H-^{13}C Heteronuclear Multiple Bond Correlation (HMBC) Spectroscopy for carajurin

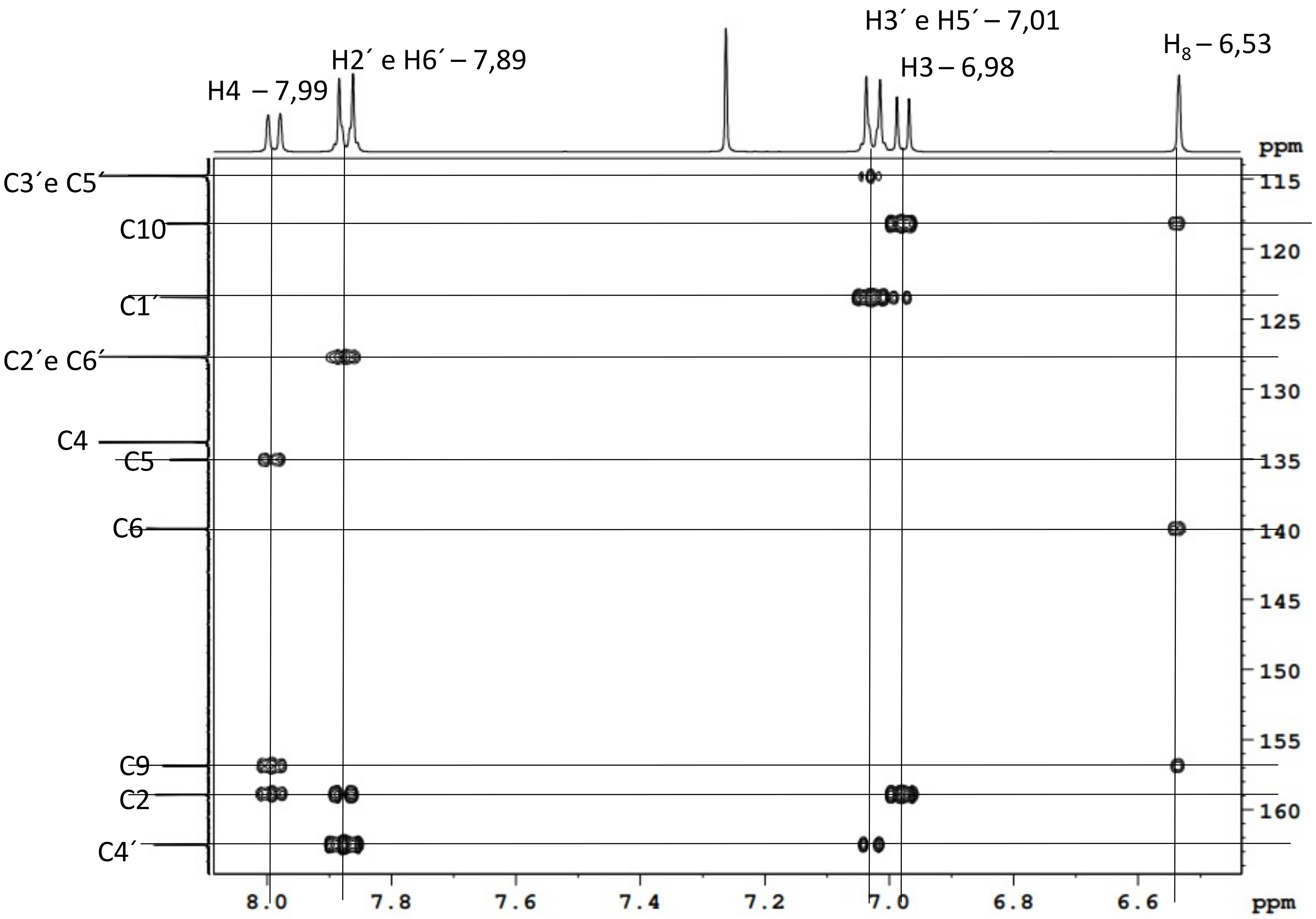

Figure S3 –^{1}H-[13]C Heteronuclear Single Quantum Correlation (HSQC) Spectroscopy for carajurin

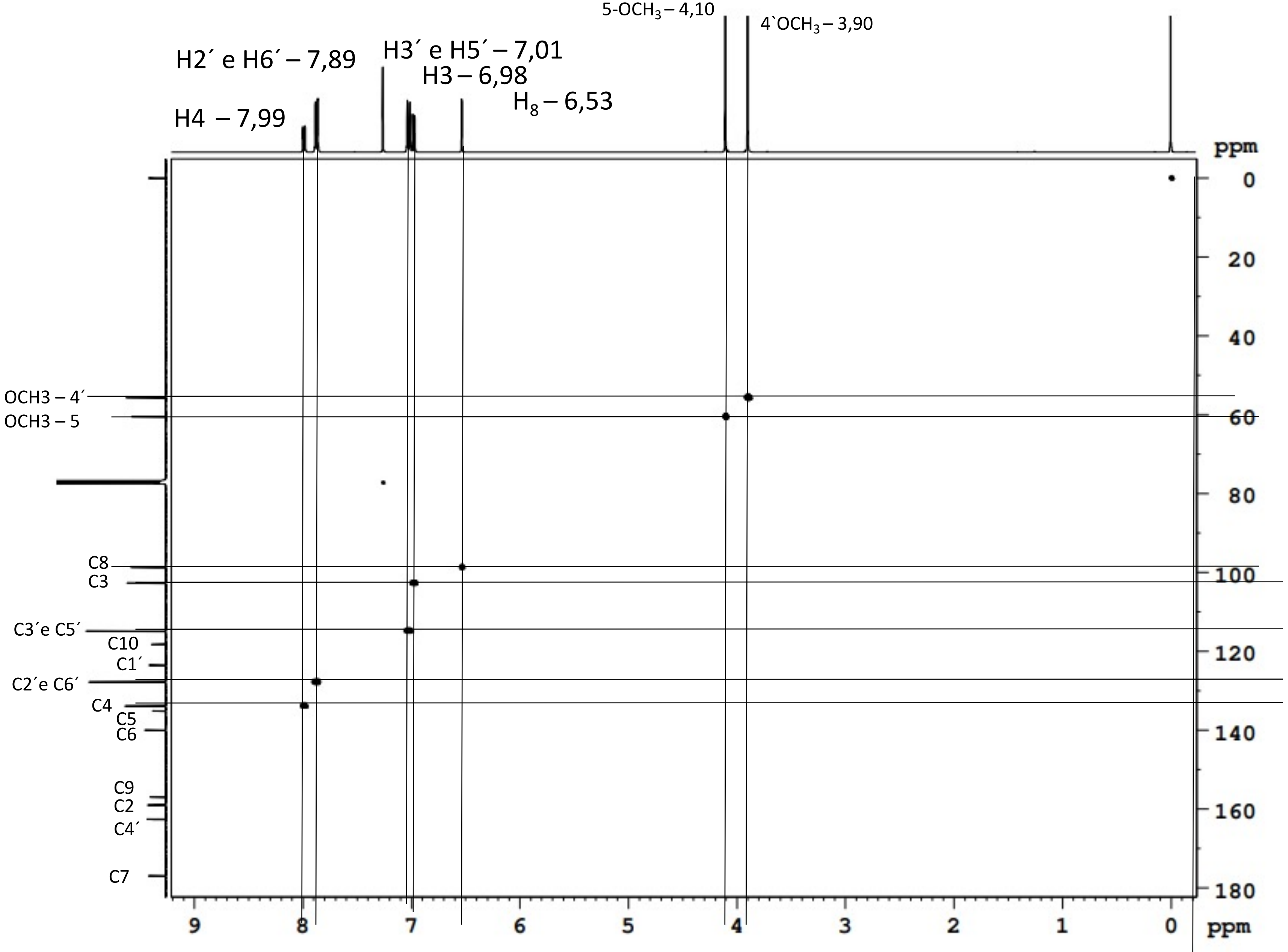

Figure S4 –^{1}H-^{1}H Homonuclear Correlation (COSY) Spectroscopy for carajurin

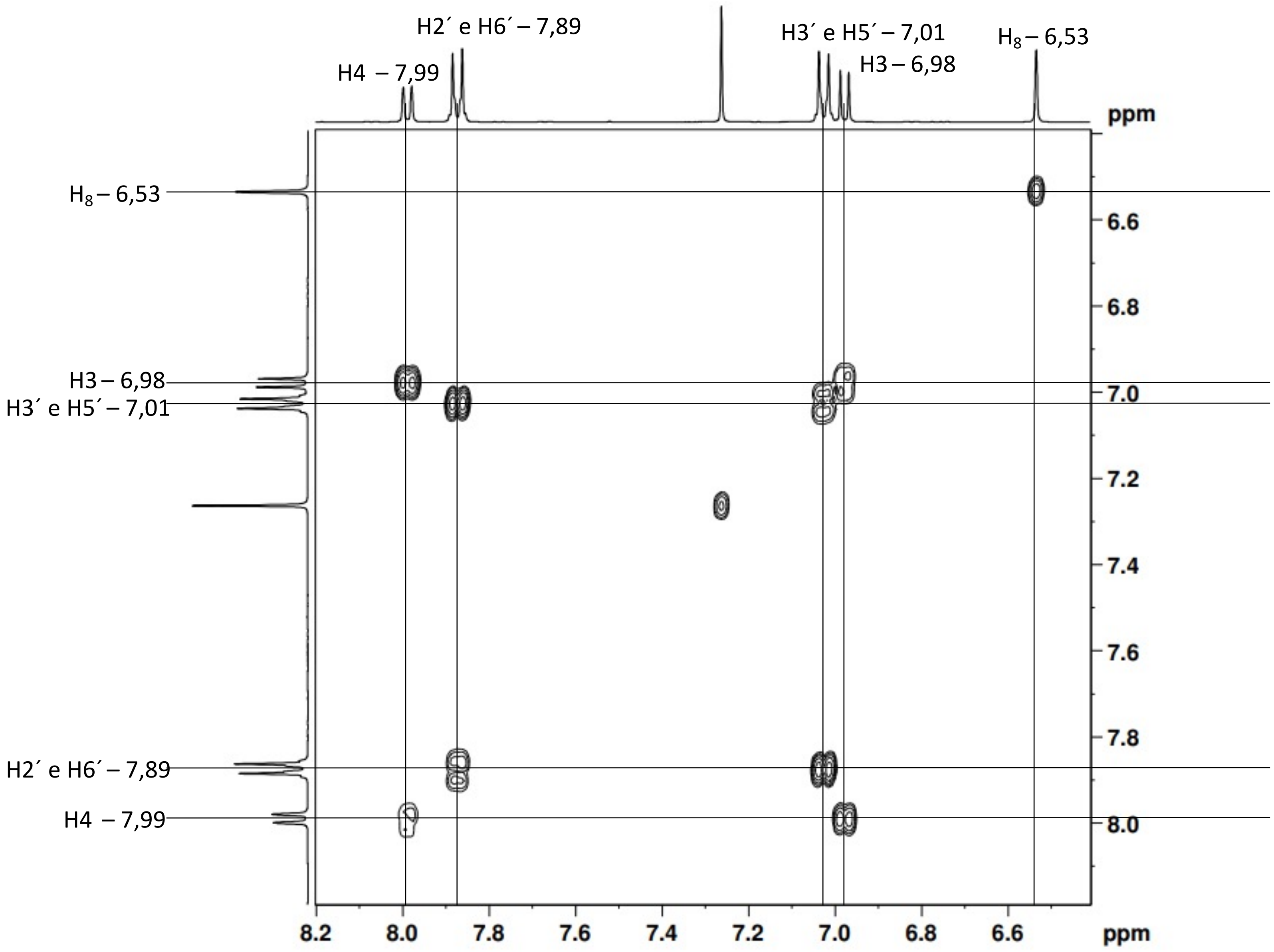

Supplement: Supplementary file 1 [file pharmaceuticals-15-00331-s001.zip › pharmaceuticals-1587026-supplementary.pdf]
